# Quantitative Evaluation of Gonioscopic and EyeCam Assessments of Angle Dimensions Using Anterior Segment Optical Coherence Tomography

**DOI:** 10.1167/tvst.7.6.33

**Published:** 2018-12-27

**Authors:** Benjamin Y. Xu, Anmol A. Pardeshi, Bruce Burkemper, Grace M. Richter, Shan C. Lin, Roberta McKean-Cowdin, Rohit Varma

**Affiliations:** 1 USC Roski Eye Institute, Department of Ophthalmology, Keck School of Medicine at the University of Southern California, Los Angeles, CA, USA; 2 Beckman Vision Center, Department of Ophthalmology, University of California San Francisco, San Francisco, CA, USA; 3 Department of Preventive Medicine, Keck School of Medicine at the University of Southern California, Los Angeles, CA, USA

**Keywords:** primary angle closure glaucoma, anterior segment optical coherence tomography, ocular imaging, angle closure, gonioscopy

## Abstract

**Purpose:**

To evaluate the relationship between angle dimensions assessed by gonioscopy or EyeCam and anterior segment optical coherence tomography (AS-OCT).

**Methods:**

Subjects aged 50 years or older were recruited from the Chinese American Eye Study (CHES). Each subject underwent a complete ocular exam, including gonioscopy, AS-OCT, and EyeCam. Angle closure was defined as three or more quadrants in which pigmented trabecular meshwork could not be visualized. Angle opening distance (AOD), angle recess area (ARA), trabecular iris space area (TISA), trabecular iris angle (TIA), and scleral spur angle (SSA) were measured in each AS-OCT image.

**Results:**

709 eyes (272 angle closure, 437 open angle) from 709 subjects were analyzed. Mean gonioscopy and EyeCam grades tended to increase as AS-OCT measurements increased. There were strong correlations overall between AS-OCT measurements and gonioscopy (*r* > 0.73) and EyeCam (*r* > 0.68) grades. However, correlations with AS-OCT measurements were weak for gonioscopy (*r* < 0.38) and EyeCam (*r* < to 0.27) among eyes with angle closure. Mean AS-OCT measurements differed for eyes with Shaffer grade 0 in all four quadrants among eyes with varying degrees of angle closure on gonioscopy (*P* < 0.01) but did not differ among eyes with varying degrees of angle closure on EyeCam (*P* > 0.27).

**Conclusions:**

Angle assessments by gonioscopy and EyeCam are weakly related to angle dimensions in eyes with angle closure.

**Translational Relevance:**

AS-OCT imaging raises concerns about current clinical methods that rely on direct visualization of ACA structures to assess the degree of angle closure.

## Introduction

Primary angle closure glaucoma (PACG) is a common cause of permanent visual impairment and blindness worldwide.^1^ PACG is the most severe form of primary angle closure disease (PACD), a spectrum of diseases characterized by appositional or synechial closure of the anterior chamber angle (ACA) by the peripheral iris. Angle closure leads to impaired aqueous humor outflow through the trabecular meshwork (TM) and elevated intraocular pressure (IOP), a strong risk factor for glaucoma. Thus, accurate assessment of angle dimensions is crucial to determining the degree of angle closure and risk for developing PACG.

Gonioscopy and EyeCam (Clarity Medical Systems, Pleasanton, CA) are contact methods for directly visualizing and evaluating the ACA and its structures. Gonioscopy is the current clinical standard for assessing angle dimensions and making the diagnosis of PACD. However, gonioscopy is highly subjective and expertise-dependent, relying on the examiner's ability to identify specific anatomic landmarks. Gonioscopy also is associated with high inter-observer variability and is poorly predictive of which patients with early PACD will progress to PACG, even when performed by an experienced glaucoma specialist.^2,3^ The EyeCam is a wide-field camera capable of taking photographs of the ACA. EyeCam imaging can be performed by a trained technician rather than a physician and shows good agreement with gonioscopy for detection of angle closure.^4,5^ However, it is unclear how effectively gonioscopy or Eyecam can quantify the degree of angle closure in the absence of other clinical signs, such as peripheral anterior synechiae (PAS) and elevated IOP.

Anterior segment optical coherence tomography (AS-OCT) is a noncontact imaging method that acquires cross-sectional images of the anterior chamber and its structures by measuring their optical reflections.^6^ In contrast to gonioscopy and EyeCam, AS-OCT imaging is an objective and quantitative assessment method.^7,8^ In addition, modern AS-OCT devices produce precise measurements with excellent intra and interuser reproducibility compared to earlier time-domain devices.^2,9–14^ AS-OCT parameters have been evaluated in terms of their ability to detect eyes with gonioscopic angle closure.^12,15^ However, limited work exists evaluating how well gonioscopy or EyeCam assessments are able to quantify angle dimensions measured by AS-OCT. Our study uses population-based AS-OCT data on Chinese American subjects to characterize the relationship between semiquantitative gonioscopic or EyeCam grades and quantitative AS-OCT measurements in eyes with and without angle closure.

## Methods

Subjects were recruited from the Chinese American Eye Study (CHES), a population-based, cross-sectional study that included 4582 Chinese participants aged 50 years and older residing in the city of Monterey Park, California. Ethics committee approval was obtained previously from the University of Southern California Medical Center institutional review board. All study procedures adhered to the recommendations of the Declaration of Helsinki.

Inclusion criteria for the study included CHES subjects who underwent examination by gonioscopy, AS-OCT, and EyeCam under standardized dark lighting conditions. Exclusion criteria included history of prior eye surgery (e.g., cataract extraction, corneal transplant, incisional glaucoma surgery, retina surgery), penetrating eye injury, or the presence of corneal disorders that precluded visualization of angle structures. Subjects with history of laser peripheral iridotomy (LPI) were not excluded. In subjects with bilateral angle closure or open angles, one eye was selected randomly for analysis. In subjects with unilateral angle closure, the angle closure eye was selected for analysis. Angle closure was defined as any eye in which the pigmented TM could not be visualized in three or more quadrants (greater than 270 degrees) of the angle. This definition was the same for gonioscopy and EyeCam.

### Clinical Assessment

As participants of CHES, each subject underwent a complete ocular examination including gonioscopy, AS-OCT imaging, and EyeCam imaging.^16^ Clinical examinations were performed by two glaucoma-trained ophthalmologists (DW, CLG).

Gonioscopy was performed with a Posner-type 4-mirror lens (Model ODPSG; Ocular Instruments, Inc., Bellevue, WA) under dark ambient lighting conditions (0.1 cd/m^2^) by two trained ophthalmologists (DW, CLG) masked to other examination findings. A 1-mm light beam was reduced to a narrow slit. Care was taken to avoid light from falling on the pupil and to avoid inadvertent indentation during examination. The gonioscopy lens could be tilted to gain a view of the angle over the convexity of the iris. The angle in each quadrant was graded using the modified Shaffer grading system based on identification of anatomic landmarks: grade 0, no structures visualized; grade 1, nonpigmented TM visible; grade 2; pigmented TM visible; grade 3, scleral spur visible; grade 4, ciliary body visible.

### AS-OCT Imaging and Image Analysis

AS-OCT imaging was performed before pupillary dilation under dark ambient lighting conditions by a single trained ophthalmologist (DW) with the Tomey CASIA SS-1000 swept-source Fourier-domain device (Tomey Corporation, Nagoya, Japan). A total of 128 two-dimensional cross-sectional AS-OCT images were acquired per eye. During imaging, the eyelids were gently retracted taking care to avoid inadvertent pressure on the globe.

Raw image data were imported into the Tomey SS-OCT Viewer software (version 3.0, Tomey Corporation, Nagoya, Japan) which automatically segmented anterior chamber structures and produced measurements of various AS-OCT parameters once the scleral spurs were marked. Four images, equivalent to eight sections through the angle, were analyzed per eye to capture the majority of anatomic variation in ACA.^17^ The first image analyzed was oriented along the horizontal (temporal-nasal) meridian. Additional OCT images were evenly spaced 45° apart from the horizontal meridian. The data were exported as an Excel format data file.

One observer (AAP) masked to the identities and examination results of the subjects manually confirmed the structural segmentation and marked the scleral spurs in each image. The scleral spur was defined as the inward protrusion of the sclera where a change in curvature of the corneoscleral junction was observed.^18^ Eyes that were not imaged under dark ambient lighting conditions, had corrupt images, or had three or more scleral spurs that could not be identified were excluded from the analysis.

Data from 10 anterior segment parameters describing the dimensions of the angle were analyzed: angle opening distance (AOD), angle recess area (ARA), trabecular iris space area (TISA), trabecular iris angle (TIA), and scleral spur angle (SSA) measured at 500 and 750 um from the scleral spur. Measurement values from the four cross-sectional images were averaged to produce a single mean measurement value. Intraobserver reproducibility of measurements was calculated in the form of intraclass correlation coefficients (ICCs). ICCs were calculated for each parameter based on images from 20 open angle and 20 angle closure eyes graded three months apart. All data analyses were performed using MATLAB (Mathworks, Natick, MA).

### EyeCam Imaging and Image Grading

EyeCam imaging was performed with the subject in the supine position under dark ambient lighting conditions by a single trained technician. Topical anesthetic drops (proparacaine hydrochloride 0.5%; Alcon Laboratories, Inc., Fort Worth, TX) and a coupling gel were applied to the eye. Images were obtained from all four quadrants (inferior, superior, nasal, and temporal quadrants sequentially) of both eyes. Care was taken to avoid contact between the imaging probe and the cornea and potential compression of the eye. If the view of the angle was blocked by a convex iris curvature, the technician was allowed to rotate the probe tip up to 10° anteriorly along the cornea to better visualize the angle.

EyeCam images were uploaded to a password-protected online data storage system. Images were graded by a single glaucoma trained specialist (SCL) masked to other examination findings. Parameters assessed included image quality and angle grade by structures identified. Image quality was graded between 1 and 3, with grade 1 representing a clear image, grade 2 a slightly blurred image with distinguishable angle structures, and grade 3 a blurry image with indistinguishable angle structures. Angle grading was based on the identification of anatomic landmarks: grade 0, no structures visualized; grade 1, nonpigmented TM visible; grade 2; pigmented TM visible; grade 3, scleral spur visible; grade 4, ciliary body visible. These angle grading categories matched the Shaffer classification system used to grade the eyes clinically on gonioscopy.

### Statistical Analyses

Mean gonioscopy grades were calculated by averaging the numerical grades (0–4) from all four quadrants and ranged from 0 to 4 in increments of 0.25. Mean and standard deviation (SD) of AS-OCT measurements were calculated for each mean gonioscopy grade. Mean AS-OCT measurements corresponding to each mean gonioscopy grade were compared using analysis of variance (ANOVA) and with Tukey honest significance difference tests for multiple comparisons. Mean AS-OCT measurements that differed significantly (*P* < 0.05) from at least two others were considered as having different mean AS-OCT measurements overall. All analyses were conducted at the significance level of 0.05.

The relationship between gonioscopy or EyeCam grades and AS-OCT measurements was calculated for the subset of eyes that fit the definition of angle closure and for all eyes in the population (see Table 2). The distribution of the data points was assessed for normality using the Kolmogorov-Smirnov (K-S) test. Spearman correlation coefficients and their *P* values were calculated for each set of grades and measurements.

## Results

Of 846 eyes from 846 subjects recruited from CHES for this study, 137 were excluded due to a history of intraocular surgery (13), incomplete or corrupt AS-OCT imaging data (55), poor quality of three or more AS-OCT images (24), or poor quality of one or more EyeCam images (45). Therefore, 709 eyes from 709 subjects (272 consecutive with angle closure and 437 consecutive with open angles; mean age 60.8 ± 7.7 years; range 50–91; 230 [32.4%] male, 479 [67.6%] female) were included in the analyses (Table 1). Mean IOP, gonioscopy grade, and central corneal thickness (CCT) were 15.5 ± 3.1 mmHg (range, 8.0–35.66), 2.1 ± 1.3 (range, 0–4.0), and 560.0 ± 33.3 μm (range, 464.3–673.9 μm), respectively.

**Table 1:**
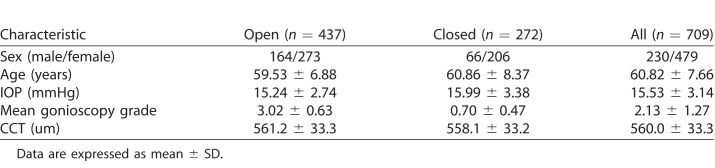
Characteristics of Open Angle, Angle Closure, and All Subjects

**Table 2:**
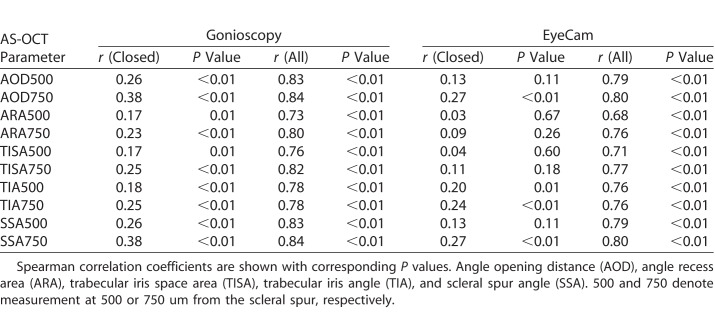
Correlation Between AS-OCT Measurements and Gonioscopy or EyeCam Grades

Intraobserver ICC values for observer AAP reflected excellent measurement reproducibility for all parameters. The ICC values were: AOD500, 0.90; AOD750, 0.96; ARA500, 0.86; ARA750, 0.91; TISA500, 0.89; TISA750, 0.92; TIA500, 0.82; TIA750, 0.94; SSA500, 0.90; and SSA750, 0.94.

### Relationship between AS-OCT Measurements and Gonioscopy or EyeCam Grades

**Figure 1:**
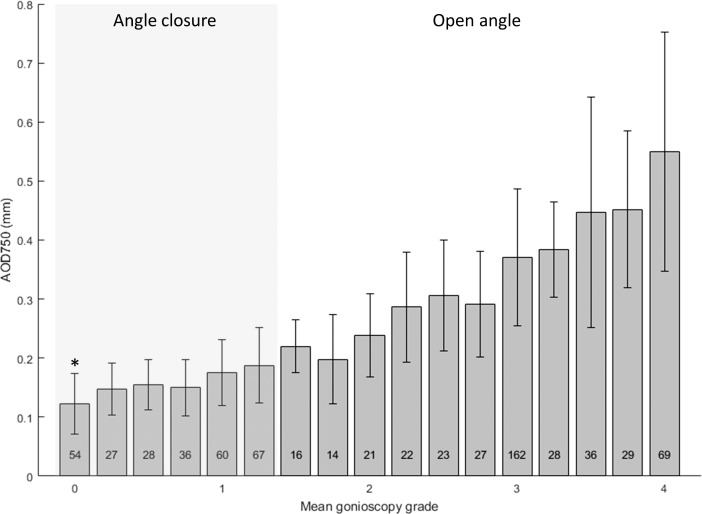
Relationship between mean AOD750 measurement and mean gonioscopy grade for all eyes. Error bars: Indicate SD of AOD750 measurements. Grey box indicates eyes that fit definition of angle closure (pigmented TM not visualized in three or more quadrants). Numbers indicate number of subjects in each group. *Indicates group of angle closure eyes for which mean AOD750 measurements differs significantly from that of other angle closure eyes (ANOVA, P < 0.01; Tukey HSD < 0.01).

Mean AS-OCT measurements tended to increase as mean gonioscopic grades increased for AOD750 (Fig. 1) and for all other AS-OCT parameters. There was a significant difference in mean AS-OCT measurements among the six groups of eyes (*n* = 272 eyes) with varying degrees of angle closure on gonioscopy (ANOVA; *P* < 0.01). The numbers of eyes in each group ranged from 27 to 67. Mean AS-OCT measurement of eyes with the most severe angle closure (Shaffer grade 0 in all quadrants, mean gonioscopy grade = 0) differed significantly (Tukey pairwise; *P* < 0.003) from mean AS-OCT measurements of eyes with less severe angle closure. The remaining five groups of eyes (mean gonioscopy grade = 0.25–1.25) did not differ from each other (Tukey pairwise; *P* > 0.05). This was true for all 10 AS-OCT parameters.

**Figure 2:**
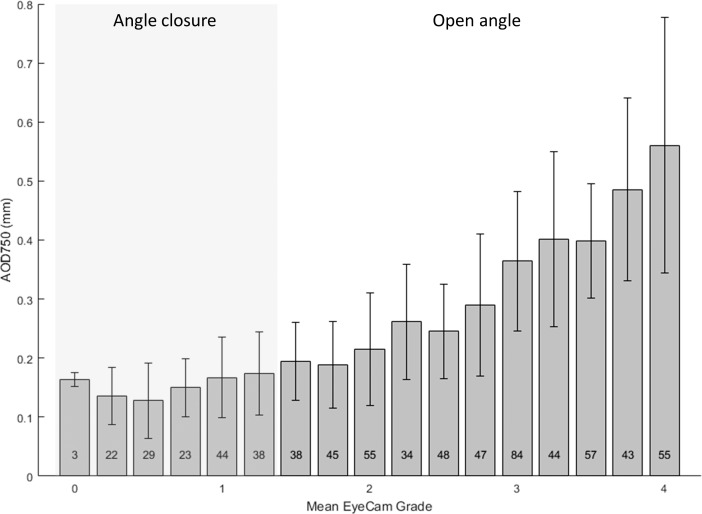
Relationship between mean AOD750 measurements and mean EyeCam grades for all eyes. Conventions same as for Figure 1. Mean AOD750 did not differ significantly among groups of angle closure eyes (ANOVA, P = 0.27; Tukey HSD > 0.05).

Mean AS-OCT measurements also tended to increase as mean EyeCam grades increased for all AS-OCT parameters (Fig. 2). However, there was no significant difference in mean AS-OCT measurements among the six groups of eyes (*n* = 159 eyes) with varying degrees of angle closure on EyeCam (ANOVA; *P* > 0.27). The numbers of eyes in each group ranged from three to 44.

### Correlation between AS-OCT Measurements and Gonioscopy or EyeCam Grades

There was a strong correlation between AS-OCT measurements and gonioscopy grades among all eyes (Table 2). The correlation was strongest for AOD750 and SSA750 (*r* = 0.84 for both) and weakest for ARA500 (0.73). However, there was only weak to moderate correlation between AS-OCT measurement values and gonioscopy grades among eyes with angle closure. The correlation was strongest for AOD750 and SSA750 (*r* = 0.41) and weakest for ARA500 (*r* = 0.26). This relationship was significant (*P* < 0.01) for all parameters across the entire population and the subset of eyes with angle closure.

There was a strong correlation between AS-OCT measurements and EyeCam grades among all eyes (Table 2). The correlation was strongest for AOD750 and SSA750 (*r* = 0.80) and weakest for ARA500 (0.68). However, there was only weak-to-moderate correlation between AS-OCT measurements and gonioscopy grades among eyes with angle closure. The correlation was strongest for AOD750 and SSA750 (*r* = 0.27) and weakest for ARA500 (*r* = 0.03). This relationship was significant (*P* < 0.01) for all parameters across the entire population and for AOD750, TIA500, TIA750, and SSA750 across the subset of eyes with angle closure.

## Discussion

In this cross-sectional study, we used AS-OCT measurements to perform a quantitative analysis of gonioscopic and EyeCam assessments of angle dimensions in a large population-based cohort of Chinese Americans. AS-OCT measurements and gonioscopy or EyeCam grades were well correlated when compared across eyes spanning a wide range of angle dimensions. However, AS-OCT measurements were weakly correlated with gonioscopy and EyeCam grades across eyes that fit the definition of angle closure, with little or no difference in mean AS-OCT measurements between eyes with varying degrees of angle closure. To our knowledge, this is the first study that uses AS-OCT measurements to assess the ability of gonioscopy and EyeCam to quantify angle dimensions in eyes with and without angle closure. These findings raise concerns about clinical methods that rely on direct visualization of ACA structures for the diagnosis and management of patients with PACD.

PACD is divided into three discrete categories of disease: primary angle closure suspect (PACS), primary angle closure (PAC), and PACG. The majority of patients with angle closure occupy the category of PACS based on gonioscopic assessments of the ACA.^19–21^ The diagnosis of PAC relies on additional exam findings, such as PAS and elevated IOP that are not direct measures of angle dimensions. This categorization system is restricted by an important limitation of gonioscopy that is highlighted by our findings: gonioscopy is poorly able to quantify the degree of angle closure for the majority of patients identified with PACD. Gonioscopy grades indicated a range of angle dimensions among angle closure eyes, with mean gonioscopy grades ranging between 0 and 1.25 and individual quadrant grades ranging between 0 and 2. However, based on quantitative analysis of AS-OCT measurements, these eyes could be differentiated only into two broad categories of angle closure: high degree (Shaffer grade 0 in all quadrants) or low degree (all other permutations of grades that fit the definition of angle closure). In addition, there were only weak correlations between AS-OCT measurements and gonioscopy grades in angle closure eyes. These results suggested that gonioscopy grades may be unreliable when attempting to quantify subtle differences in angle dimensions to guide the management of angle closure patients.

The decision whether to treat or monitor a patient with gonioscopic angle closure in the absence of PAS, elevated IOP, and/or glaucoma is a challenging one. There are effective treatments that alleviate angle closure, such as LPI and lens extraction.^22^ However, there is no consensus on when to perform these treatments in patients with PACS. Progression of PACS to PAC and PACG is not uncommon.^23,24^ However, our results suggested that it could be difficult to determine appropriate monitoring schedules and detect changes in angle dimensions that mark the progression of angle closure based on gonioscopy alone. Patients with PACS and high degree closure could be at higher risk for PACG than patients with low degree closure and may benefit from earlier prophylactic treatment. However, longitudinal studies are required before our results can be ascribed clinical significance and applied to patient care.

The variable relationship between AS-OCT measurements and gonioscopy grades indicated that there may be factors apart from angle dimensions that influence gonioscopic assessments of the ACA. Part of the variability may be attributable to the role that iris curvature and lens position have in visualizing the ACA. In angle closure eyes, convex bowing of the iris and anterior positioning of the lens sometimes are present and can hinder accurate assessments of angle dimensions.^25^ In open angle eyes, these anatomic variables have a smaller role and consequently gonioscopy provides more accurate assessments of the ACA. In our study, AOD750 and SSA750 were the parameters best correlated with gonioscopic grade among all eyes and eyes with angle closure. This finding is consistent with previous work that evaluated the performance of AS-OCT parameters and found that AOD750 performed best in the detection of gonioscopic angle closure.^12,26^

One major criticism of gonioscopy is that it is examiner-dependent, even when performed under standardized conditions by experienced glaucoma specialists. Even with an ophthalmologist trained in a standardized methodology for gonioscopy, we found weak correlations between AS-OCT and gonioscopy in our study. We analyzed the relationship between AS-OCT measurements and EyeCam grades to lessen the possibility of subjectivity in grading and to validate our reported relationship between AS-OCT measurements and gonioscopy grades. EyeCam demonstrated a similar pattern of correlation with AS-OCT as gonioscopy even though visualization of ACA structures was achieved by a completely different method. One difference between the two assessment methods is that EyeCam is performed in the supine position while gonioscopy and AS-OCT are performed in the seated position. Changes in body position induce modest changes in angle dimensions, which may explain the weaker correlations between AS-OCT and EyeCam compared to gonioscopy.^4,5,27^ However, previous studies reported fair agreement between gonioscopy and EyeCam in the detection of angle closure, and the correlation results with EyeCam closely resemble our results with gonioscopy; thus, providing cross-validation of our correlation results.

Strengths of this study include a large sample size, especially of angle closure eyes. In addition, previous analysis of CHES EyeCam images found excellent inter- and intraobserver agreement in the detection of angle closure (κ = 0.82 and 0.87, respectively).^4^ However, our study has some limitations. First, we are unable to report measures of intra- and interexaminer reproducibility of gonioscopy based on data from CHES. However, prior work demonstrated good to excellent reproducibility of gonioscopy grades among trained examiners.^2^ In addition, the majority of gonioscopy (>90%) was performed by a single examiner (DW). Second, the number of subjects detected with severe angle closure (Shaffer grade 0 in all quadrants) by EyeCam was small. With a larger sample size, mean AS-OCT measurement for these eyes may have differed from other eyes with angle closure, similar to gonioscopy. Finally, weak correlations between angle grades and AS-OCT measurements could be attributed to inaccuracies of AS-OCT measurements as there currently is no gold standard device to verify these measurements. However, measurements are highly reproducible and correlated between different AS-OCT devices, which indirectly validates their quantitative performance.^28,29^

In summary, we demonstrated that gonioscopy and EyeCam are poorly able to quantify angle dimensions in eyes that fit the definition of angle closure. These findings serve as an important reminder that caution should be exercised when clinical decisions are made based on gonioscopy grades alone, especially in eyes with angle closure. These findings also support the consideration for an expanded role for AS-OCT in the clinical management of patients with angle closure detected by gonioscopy. We hope this study prompts further investigation on how clinical methods to manage patients identified with angle closure by gonioscopy can be expanded and improved.
